# When Fitness Turns Fatal: A Case of Severe Rhabdomyolysis in a 20-Year-Old Male

**DOI:** 10.7759/cureus.97811

**Published:** 2025-11-25

**Authors:** Wardah Tariq, Habeeb Kamal, Aung M Naing, Farah Shahid, Suhail Ahmed

**Affiliations:** 1 General Medicine, University Hospitals of Leicester NHS Trust, Leicester, GBR

**Keywords:** acute kidney injury, case report, creatine kinase, exercise-induced muscle injury, exertional rhabdomyolysis

## Abstract

Exertional rhabdomyolysis (ER) is a potentially life-threatening condition caused by intense or prolonged physical activity, leading to skeletal muscle injury and leakage of intracellular components into the circulation. Although commonly recognized in athletes and military recruits, it may also occur in previously healthy individuals undertaking unaccustomed high-intensity exercise. We report a 20-year-old male with no significant medical history who presented with muscle soreness, dysuria, and dark urine following increased gym activity. Laboratory studies demonstrated extremely elevated creatine kinase (CK) levels consistent with severe ER; however, kidney function remained entirely normal despite the markedly elevated CK, a feature that makes this case clinically distinctive. Prompt recognition and aggressive intravenous fluid resuscitation resulted in full recovery. This case provides educational value by emphasizing that even “sky-high” CK levels do not invariably lead to renal impairment when ER is rapidly identified and managed, underscoring the importance of early intervention and careful monitoring in individuals engaging in unsupervised intense exercise.

## Introduction

Rhabdomyolysis refers to the dissolution of skeletal muscle fibers with subsequent release of intracellular components, including creatine kinase (CK), myoglobin, electrolytes, and sarcoplasmic proteins, into the circulation [1]. It may result from trauma, immobilization, metabolic myopathies, infections, or intense physical exertion. Exertional rhabdomyolysis (ER) is increasingly recognized among athletes and individuals engaging in unsupervised high-intensity training [2,3]. Although most cases resolve with timely management, severe complications such as acute kidney injury (AKI), disseminated intravascular coagulation (DIC), and compartment syndrome can occur [4].

ER represents a clinical spectrum ranging from mild, asymptomatic enzyme elevation to life-threatening multi-organ failure. Its pathophysiology involves excessive mechanical stress, ischemia, and metabolic exhaustion of muscle fibers, resulting in sarcolemmal disruption and leakage of intracellular constituents. Myoglobin in particular contributes to renal tubular obstruction and oxidative injury, thereby precipitating AKI. With the rising popularity of high-intensity interval training, CrossFit, and extreme fitness programs, the incidence of ER among recreational gym users has increased globally, which includes the presented case [5,6,7].

This report describes a case of severe ER with extremely high CK levels but preserved renal function, highlighting an uncommon presentation. Early recognition remains crucial to prevent complications, particularly in young individuals engaging in unsupervised, high-intensity exercise.

## Case presentation

A 20-year-old male with a history of depression, not taking antidepressant medications, presented to the emergency department with a one-day history of generalized muscle soreness, dysuria, and dark-colored urine. He denied fever, trauma, recent illness, substance use, or systemic symptoms. He reported a recent, substantial increase in both frequency and intensity of gym workouts, performing unsupervised resistance and endurance exercises for up to three hours daily as a coping mechanism for low mood.

On arrival, he was afebrile with a blood pressure of 118/76 mmHg, heart rate of 82 beats per minute, and oxygen saturation of 99% on room air. Examination revealed mild diffuse tenderness of proximal limb muscles without swelling, weakness, or compartmental features. No neurological deficits were observed. Urinalysis showed dark urine with positive blood and mild proteinuria but no casts. Laboratory testing demonstrated a markedly elevated CK level of 128,986 IU/L, an elevated alanine aminotransferase (ALT) of 142 IU/L, normal creatinine of 0.9 mg/dL, normal inflammatory markers, normal complete blood count, and electrolytes within normal limits, including potassium, which ranged between 3.7 and 4.4 on various occasions throughout the hospital stay. Other liver function tests, including aspartate aminotransferase (AST), were elevated in a pattern consistent with muscle injury rather than intrinsic hepatic disease. Electrocardiogram, chest radiograph, and abdominal ultrasound were unremarkable, and there was no evidence of hemolysis or autoimmune pathology. All laboratory values are summarized in Table 1.

**Table 1:** Timeline of key laboratory findings and management

Hospital Day	Creatine kinase (IU/L)	Creatinine (mg/dL)	Alanine aminotransferase (IU/L)	Intervention / notes
Day 1	128,986	0.9	142	Initiated IV hydration
Day 2	89,540	0.8	130	Continued hydration; monitored urine output
Day 3	45,312	0.8	120	Symptom improvement
Day 4	15,780	0.9	100	CK trending down
Day 5	780	0.9	80	Discharged; symptom-free

**Figure 1:**
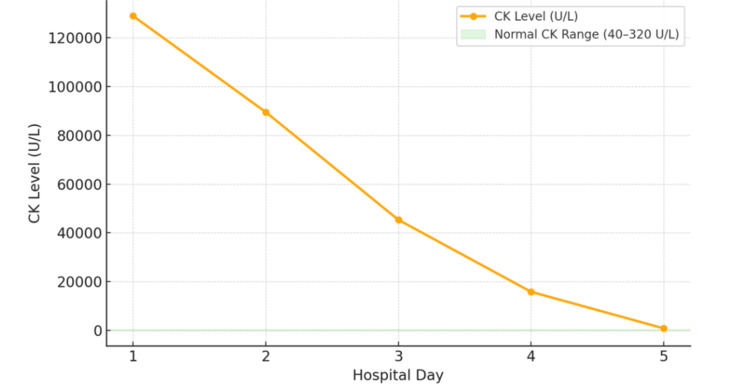
Trend of creatinine kinase over the length of hospital stay. The image was created by the authors using Canva (Canva Pty Ltd., Australia).

A diagnosis of ER was made, and the patient was admitted for aggressive intravenous hydration. He received alternating 0.9% normal saline and Hartmann’s solution at 250-300 mL/hour, with a total infusion volume of approximately 32 over four days, targeting a urine output >200 mL/hour. Urine alkalinization was not required due to stable renal function. Serial CK measurements showed a rapid decline, and creatinine remained normal throughout admission, demonstrating preserved renal function despite the extremely elevated CK level, as demonstrated in Figure 1.

The patient’s symptoms improved steadily, and he remained hemodynamically stable with no electrolyte abnormalities or evolving complications. He was discharged on day five with normalization of biochemical parameters. At two-week follow-up, CK and liver enzyme levels were within normal limits, and he was counselled on safe, graded return to exercise.

This case highlights how prompt and adequate fluid resuscitation can prevent AKI even in severe ER with exceptionally high CK levels.

## Discussion

This case adds to the growing number of reports of ER in young gym users performing unsupervised high-intensity exercise. This discussion focuses on interpreting our findings in the context of existing literature and highlighting the unique aspects of this presentation. ER occurs when strenuous or unaccustomed exercise leads to skeletal muscle breakdown with subsequent release of intracellular components, including CK and myoglobin, into the circulation [1]. Although classically associated with myalgia, weakness, and myoglobinuria [4], its severity varies greatly. A systematic review of 772 athletes identified running (54.3%) and weightlifting (14.8%) as the most common triggers, with mean CK levels of 31,481 IU/L [2]. Another review of endurance events reported ER in 43.5% of cases, with AKI occurring in 16.3%, particularly among ultra-endurance runners [3].

Our patient’s CK level of 128,986 IU/L falls among the highest values reported in the literature. Importantly, despite this extremely elevated CK, he did not develop AKI or electrolyte derangements. Prior studies consistently show that CK level alone is not a reliable predictor of AKI risk, and no definitive CK threshold universally correlates with renal injury [8,9]. Instead, host factors, including baseline hydration, timely fluid resuscitation, and absence of nephrotoxic exposures, play a greater role in determining renal outcomes. Similar cases describe patients with CK levels exceeding 100,000 IU/L who avoided AKI when aggressive hydration was implemented early [10]. This reinforces the clinical relevance of our case: remarkably high CK does not inevitably imply renal dysfunction when prompt management is instituted.

Complications of ER such as AKI, DIC, compartment syndrome, and electrolyte abnormalities (hypocalcemia and hyperkalemia being the most frequent) are well documented, with 10-30% of cases progressing to AKI [4,11]. The absence of such complications in our patient further underscores the protective value of immediate IV fluid therapy and early recognition.

Clinically, this case highlights important implications for primary care, emergency medicine, and sports medicine. With the rise of unsupervised high-intensity workouts, clinicians should maintain a high index of suspicion for ER in young individuals presenting with muscle pain and dark urine after strenuous exercise. Preventive strategies such as gradual conditioning, adequate hydration, education on warning symptoms, and supervised training are essential to reducing risk.

In sum, this case illustrates that even exceptionally high CK levels in ER may not lead to renal injury when managed promptly. Early hydration and increased awareness among gym participants and healthcare providers remain key to preventing life-threatening complications of ER.

## Conclusions

ER is an important differential in young patients presenting with muscle pain and dark urine following strenuous activity. Although often benign with timely treatment, delayed recognition can result in severe complications, including AKI and life-threatening electrolyte disturbances. This case reinforces that early diagnosis, prompt intravenous hydration, and vigilant monitoring of renal function are pivotal for favorable outcomes. Clinicians should educate high-risk groups, such as gym beginners and individuals self-managing stress through exercise, about progressive training regimens and warning symptoms like dark urine or severe myalgia. Incorporating preventive counseling into fitness programs may significantly reduce ER incidences and their potential complications.
